# In vitro assessment of Moringa Oleifera leaf extract’s protective impact on fibroblast cell line (WI-38) exposed to electronic cigarette liquid

**DOI:** 10.1038/s41598-025-14513-y

**Published:** 2025-08-16

**Authors:** Raneem F. Obeid, Yara Y. Mouselhy, Mona Eltaher, Radwa T. El-sharkawy

**Affiliations:** 1 Faculty of Oral and Dental Medicine, Future University in Egypt, Cairo, 11835 Egypt; 2 Faculty of Oral and Dental Medicine, Future University in Egypt, Cairo, 11835 Egypt; 3 Faculty of Business, Ain Shams University, Cairo, Egypt; 4 Faculty of Dentistry, Applied Science Private University, Amman, 11937 Radwa Jordan

**Keywords:** *Moringa Oleifera* leaves, Electronic cigarettes e-liquid, WI-38 cells, Apoptosis, Cell cycle, Biotechnology, Cancer, Cell biology, Drug discovery, Plant sciences

## Abstract

*Moringa oleifera* (MO), a medicinal herb, has been studied in recent decades for its diverse range of biological activities. Several studies have revealed that MO leaf extract (MOLE) possesses cytoprotective properties, while other studies have reported anti-proliferative potential. This study was conducted to assess the potential effect of MOLE on electronic cigarette liquid (EC e-liquid) treated human lung fibroblast cell line (WI-38). An MTT assay was performed to investigate the potential effect of EC e-liquid and MOLE on cell viability and determine the cytotoxic concentration (CC50) in WI-38 cells. Cells were treated with 190.2 µg/mL of EC e-liquid (EC group) and 33.2 µg/mL of EC e-liquid and MOLE (EC + MOLE group) for 24 h. Analysis of apoptosis and cell cycle was performed using flow cytometric assay. Moreover, the expression of apoptosis-related proteins Bax and Bcl2 was measured using ELISA.Our findings revealed that MOLE demonstrated a cytotoxic effect on EC e-liquid treated WI-38 cells through induction of apoptosis via up-regulation of Bax and down-regulation of Bcl2. Additionally, MOLE induced cell cycle arrest at the S phase. Overall, these findings indicate that MOLE is an inefficient cytoprotective agent against EC e-liquid cytotoxicity; nonetheless, it exerts a cytotoxic effect, suggesting a promising role as a valuable anti-proliferative agent.

## Introduction

Research and applications of medicinal herbs have gained popularity worldwide as an important source of drug discovery in various therapeutic domains due to their perceived safety, efficacy and low potential for resistance development^1,2^. Multiple biological activities of medicinal herbs such as anti-inflammatory^3,4^anti-oxidant^5^anti-bacterial^6^ and anti-diabetic^7^have been reported in the past few decades. In addition, several laboratory-based^8–10^ as well as epidemiological^11,12^ studies have demonstrated the anti-proliferative potential of medicinal herbs.

*Moringa oleifera* Lam. (MO) (genus: *Moringa*, family: *Moringaceae*), a perennial plant^13^also known as the “miracle tree”^14^ or “drumstick tree”^15^has been used for several years as one of the traditional medicinal sources in the treatment of many diseases. It is documented as a nutritious and affordable source of phytochemicals, including alkaloids, tannins, steroids, phenolic acids and flavonoids^16^ that are rich in iron, calcium, potassium, proteins, vitamins (mainly C and tocopherol) and β-carotene^15^. Extracts from different parts of MO including root, leaf, fruit, seed, fruit pod and flower have been recognized to have multiple biological activities such as anti-inflammatory^17^anti-oxidant^18^anti-bacterial^19^anti-diabetic^15^cytoprotective^20,21^ and anti-proliferative^18,22^.

Electronic cigarettes (ECs) were developed by the Ruyan Group (Holdings) Limited in Beijing, China in 2003. They were initially marketed as a safer alternative to traditional cigarettes, consequently, their use has rapidly gained popularity since their debut in 2006 in the USA and Europe^23^. While the direct harmful effects of cigarette smoking on the lungs are well documented, research on the biological effects of EC products remains limited^24^. This lack of information, despite its acknowledged importance, has led to ongoing debates within healthcare and regulatory bodies. Consequently, conflicting advice regarding the appropriate use of ECs continues to circulate^25^.

The cartridges of ECs are refilled with a refill fluid known as e-juice or e-liquid. This e-liquid contains flavoring, nicotine, and a humectant(s), such as propylene glycol (PG) and/or vegetable glycerin (VG). It is readily available, usually from third-party vendors in shopping malls or on the internet^26^.

The present study was conducted to investigate the effect of MOLE and its mechanism of action on EC e-liquid treated WI-38 cells.

## Results

### Evaluation of cytotoxicity against WI-38 cell line

#### Cytotoxicity analysis

**Fig. 1:**
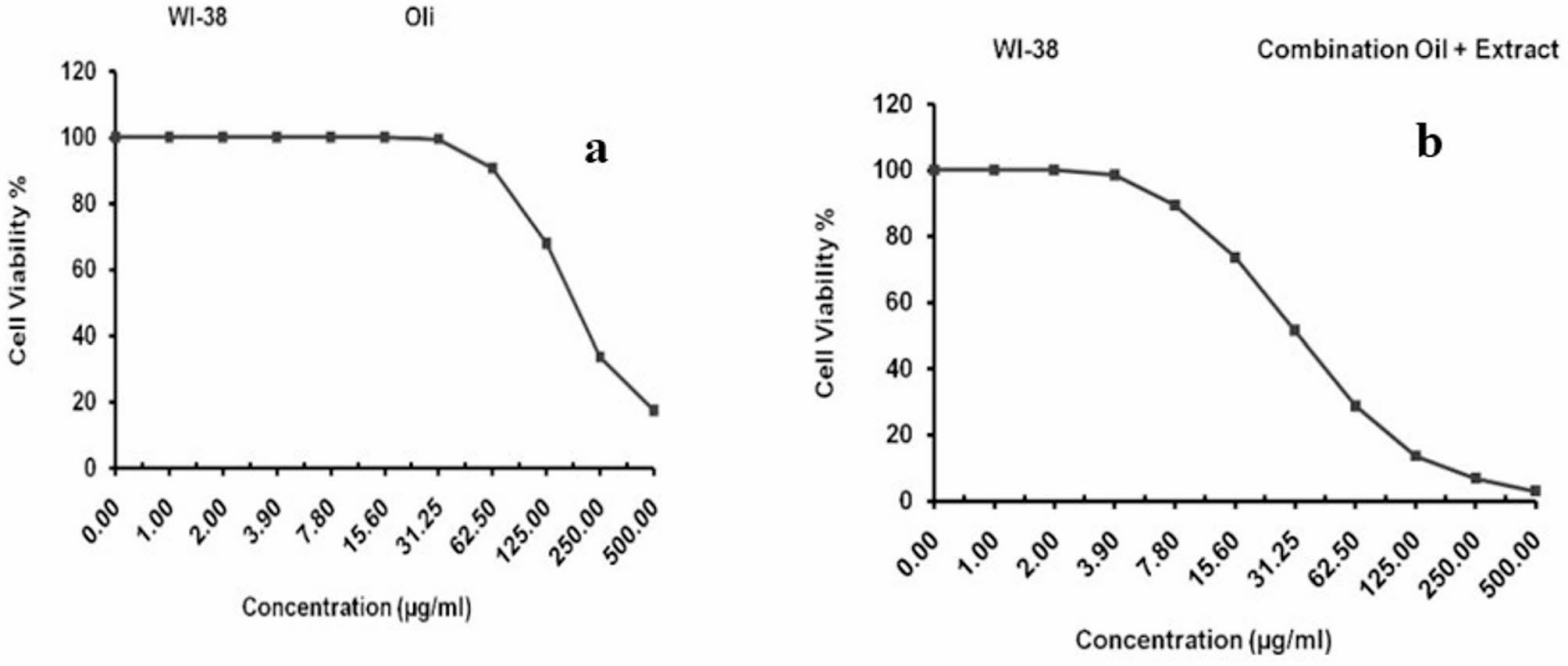
Inhibitory activity and cytotoxic concentrations of EC group **(a)** and EC + MOLE group **(b)**.

MTT assay was performed to evaluate the optimal concentration of anti-proliferative effect in EC and EC + MOLE groups. The cell lines were treated for 24 h with 500 µg/mL, 250 µg/mL, 125 µg/mL, 62.5 µg/mL, 31.25 µg/mL, 15.6 µg/mL and 7.8 µg/mL concentrations **(**Table 1**).** The CC_50_ was estimated from graphic plots of the dose response curve for each concentration using GraphPad Prism software (San Diego, CA. USA)^27^. The CC_50_ results in EC and EC + MOLE groups were 190.2 _±_ 5.17 µg/mL and 33.2 ± 1.23 µg/mL, respectively **(**Fig. 1**).** These concentrations were used for the cell culture treatment.

**Table 1:** Viability and inhibitory percentages in different sample concentrations in the experimental groups.

Sample conc.	500 (µg/ml)		250 (µg/ml)		125 (µg/ml)		62.5 (µg/ml)		31.25 (µg/ml)		15.6 (µg/ml)		7.8 (µg/ml)	
Group	EC	EC + MOLE	EC	EC + MOLE	EC	EC + MOLE	EC	EC + MOLE	EC	EC + MOLE	EC	EC + MOLE	EC	EC + MOLE
Viability %	17.43	2.86	33.64	6.79	67.85	13.47	90.63	28.76	99.42	51.38	100	73.54	100	89.21
Inhibitory %	82.57	97.14	66.36	93.21	32.15	86.53	9.37	71.24	0.58	48.62	0	26.46	0	10.79

#### Morphological evaluation of cytotoxicity against WI-38 cell line

**Fig. 2:**
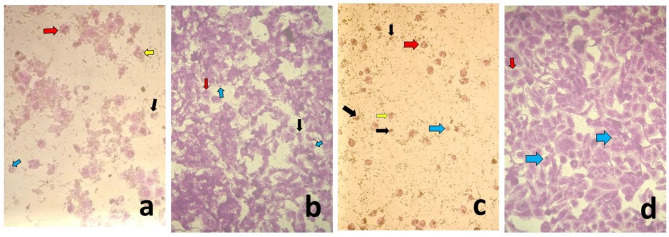
Photomicrographs of the control group **(a)**, EC group [at conc. 250 µg/mL] **(b)**, [at conc. 125 µg/mL] **(c)**, EC + MOLE group [at conc. 125 µg/mL] **(d)**, [at conc. 31.25 µg/mL] **(e)** showing: nuclear fragmentation and chromatin margination [red arrows], cellular shrinkage [black arrows], blebbing [yellow arrows] and apoptotic cells [blue arrows] (origin. mag. 100x).

Cells treated with different concentrations in EC and EC + MOLE groups, showed fewer cells and exhibited some apoptotic morphological changes, including cytoplasmic blebbing, cellular shrinkage, nuclear fragmentation, chromatin margination, and formation of apoptotic bodies (Fig. 2a, b, c, d).

### Flow cytometric analysis

#### Apoptosis analysis using flow cytometry (Annexin V-FITC/PI assay)

**Fig. 3:**
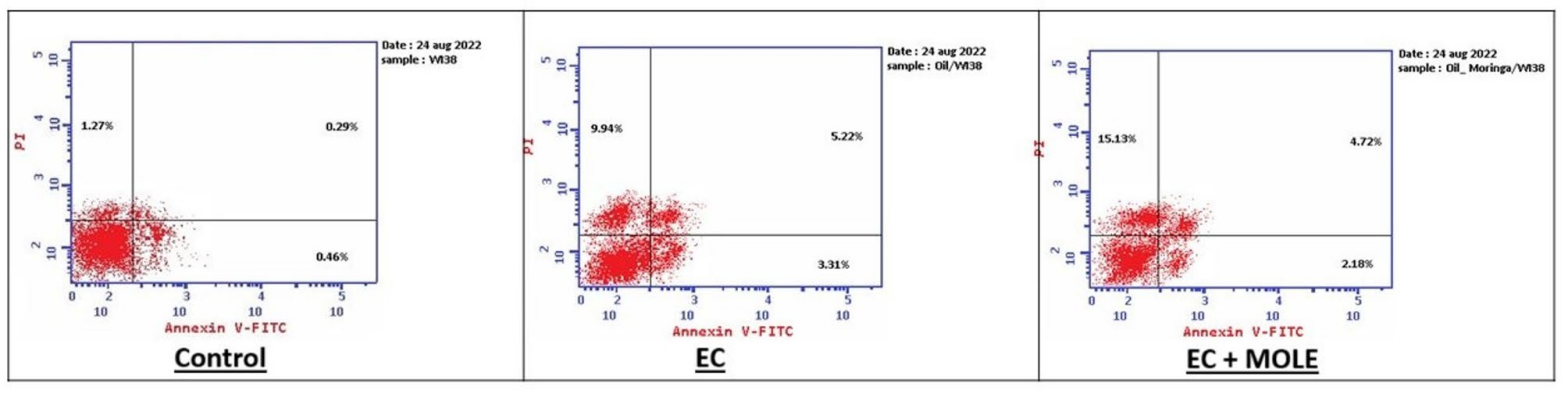
Representative Annexin V-FITC/PI dot plots of WI-38cells evaluated by flow cytometry analysis. The first quadrant represents necrotic cells (Annexin V^-^/PI ^+^), the second represents late apoptotic cells (Annexin V^+^/PI ^+^), the third represents live cells (Annexin V^-^/PI ^-^) and the fourth represents early apoptotic cells (Annexin V^+^/PI ^-^).

The double dye Annexin V-FITC/PI was used to detect the apoptotic cells in WI-38 after treatment with 190.2 µg/mL of EC e-liquid in EC group and 33.2 µg/mL of EC e-liquid and MOLE in EC + MOLE group. Results revealed that the percentage of apoptotic and necrotic cells in Control, EC and EC + MOLE groups was 2.02%, 18.47% and 22.03% respectively. Meanwhile, the percentage of early apoptotic cells was 0.46%, 3.31% and 2.18% and the percentage of late apoptotic cells was 0.29%, 5.22% and 4.72% in Control, EC and EC + MOLE groups respectively (Fig. 3).

#### Cell cycle analysis using flow cytometry

**Fig. 4:**
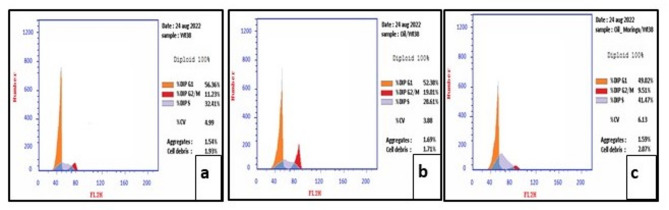
A histogram of the cell cycle analysis, showing the distribution of cells for Control group **(a)**, EC group **(b)**, and EC + MOLE group **(c)** at different phases using flow cytometry.

Flow cytometry analysis was performed to investigate the effect of 190.2 µg/mL of EC e-liquid (EC group), and 33.2 µg/mL of EC e-liquid and MOLE (EC + MOLE group) on cell cycle distribution **(**Fig. 4**).** Results showed that the highest percentage of cells was in the G0/G1 phase for all groups. Interestingly, EC + MOLE group exhibited a significant accumulation of cells (41.47%) in the S phase compared to Control group (32.41%) and EC group (28.61%). Conversely, EC group showed an increased percentage of cells (19.01%) in the G2/M phase compared to Control group (11.23%) and EC + MOLE group (9.51%). These findings suggest that EC e-liquid induced cell cycle arrest at the G2/M phase, while the combination of EC e-liquid and MOLE induced arrest at the S phase.

### ELISA

**Fig. 5:**
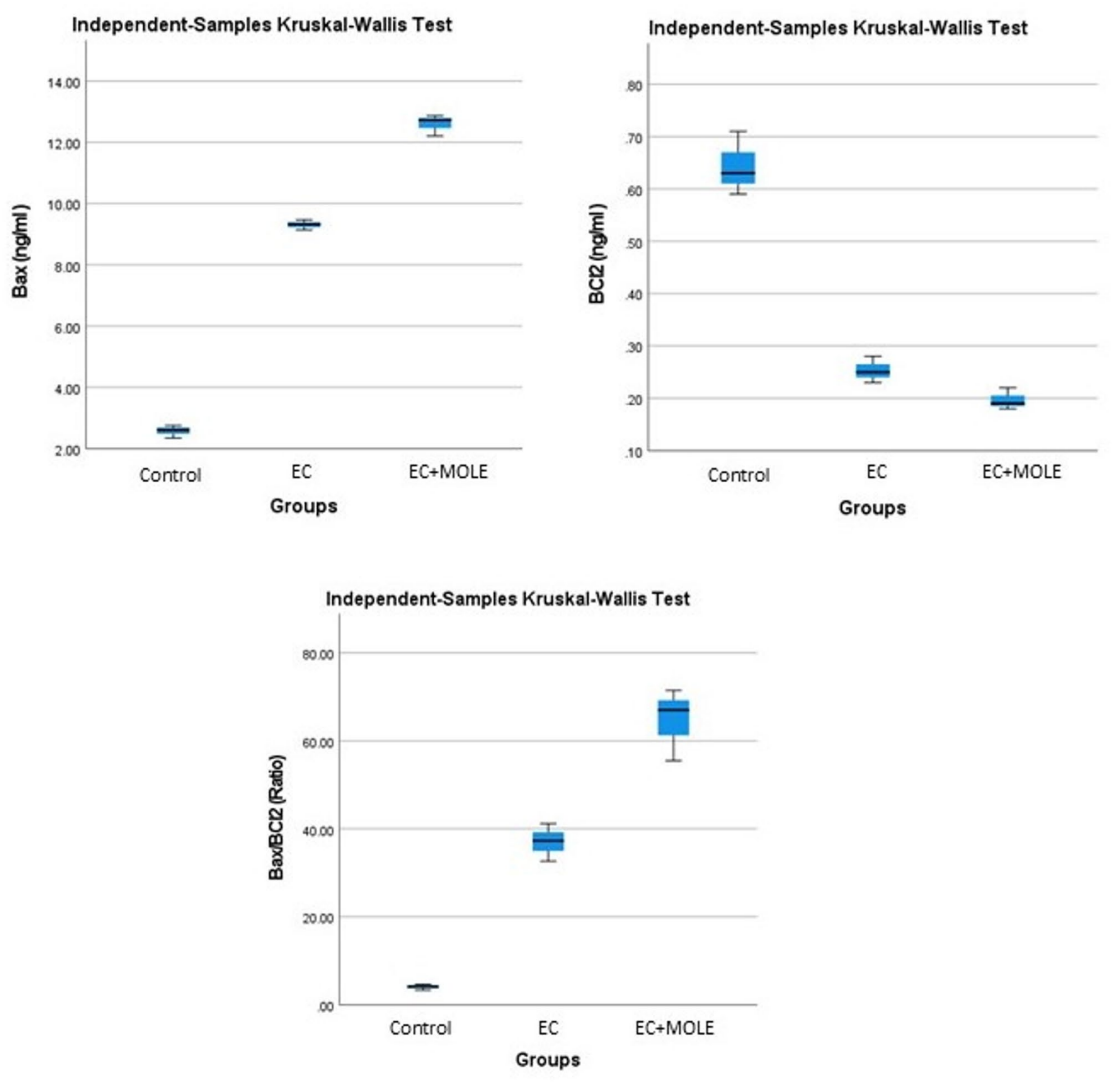
Box plots of Kruskal–Wallis representing the differences between Bax, Bcl2 and Bax/Bcl2 ratio expression in different groups.

Using ELISA technique, five samples were submitted for determination of Bax, Bcl2 and Bax/Bcl2 ratio. Regarding the expression of Bax protein, results demonstrated that EC + MOLE group showed the highest level of up-regulation of Bax protein, followed by EC group. Meanwhile, regarding the expression level of the anti-apoptotic Bcl2, EC + MOLE group showed the highest level of Bcl2 down-regulation, followed by EC group. In addition, the Bax/Bcl2 ratio results also demonstrated the highest level of expression in EC + MOLE group when compared to the other groups (Fig. 5), indicating the induction of WI-38 cells apoptosis in the mentioned groups. Moreover, the Kruskal–Wallis test demonstrated that the difference between all groups was statistically significant (Tables 2 and 3).

**Table 2:** Readings of bax, Bcl2 and Bax/Bcl2 ratio in the three groups.

Sample Code	Bax (ng/ml)			Bcl2 (ng/ml)			Bax/Bcl2 (Ratio)		
	1st	2nd	3rd	1st	2nd	3rd	1st	2nd	3rd
Control group	2.75	2.34	2.61	0.59	0.71	0.63	4.66	3.30	4.14
EC group	9.32	9.47	9.14	0.25	0.23	0.28	37.28	41.17	32.64
EC + MOLE group	12.73	12.87	12.21	0.19	0.18	0.22	67.00	71.50	55.50

**Table 3:** Descriptive statistics of significant differences in mean and standard deviation values for bax, Bcl2 and Bax/Bcl2 ratio in different groups.

Groups	Control group	EC group	EC + MOLE group	Sig.^a, b^	Decision
**Bax (ng/ml)**					
Mean ± SD	2.57 ± 0.21	9.31 ± 0.17	12.6 ± 0.35	0.027	Reject the null hypothesis
Range	0.41	0.33	0.66		
**Bcl2 (ng/ml)**					
Mean ± SD	0.64 ± 0.06	**0.25 ± 0.03**	**0.2 ± 0.02**	**0.027**	**Reject the null hypothesis**
Range	0.12	0.05	0.04		
Bax/Bcl2 (Ratio)					
Mean ± SD	4.03 ± 0.69	37.03 ± 4.27	64.67 ± 8.25	0.027	Reject the null hypothesis
Range	1.36	8.53	16		

a. *The significance level is 0.050.*

b. *Asymptotic significance is displayed.*

### Transmission electron microscope (TEM) evaluation

**Fig. 6:**
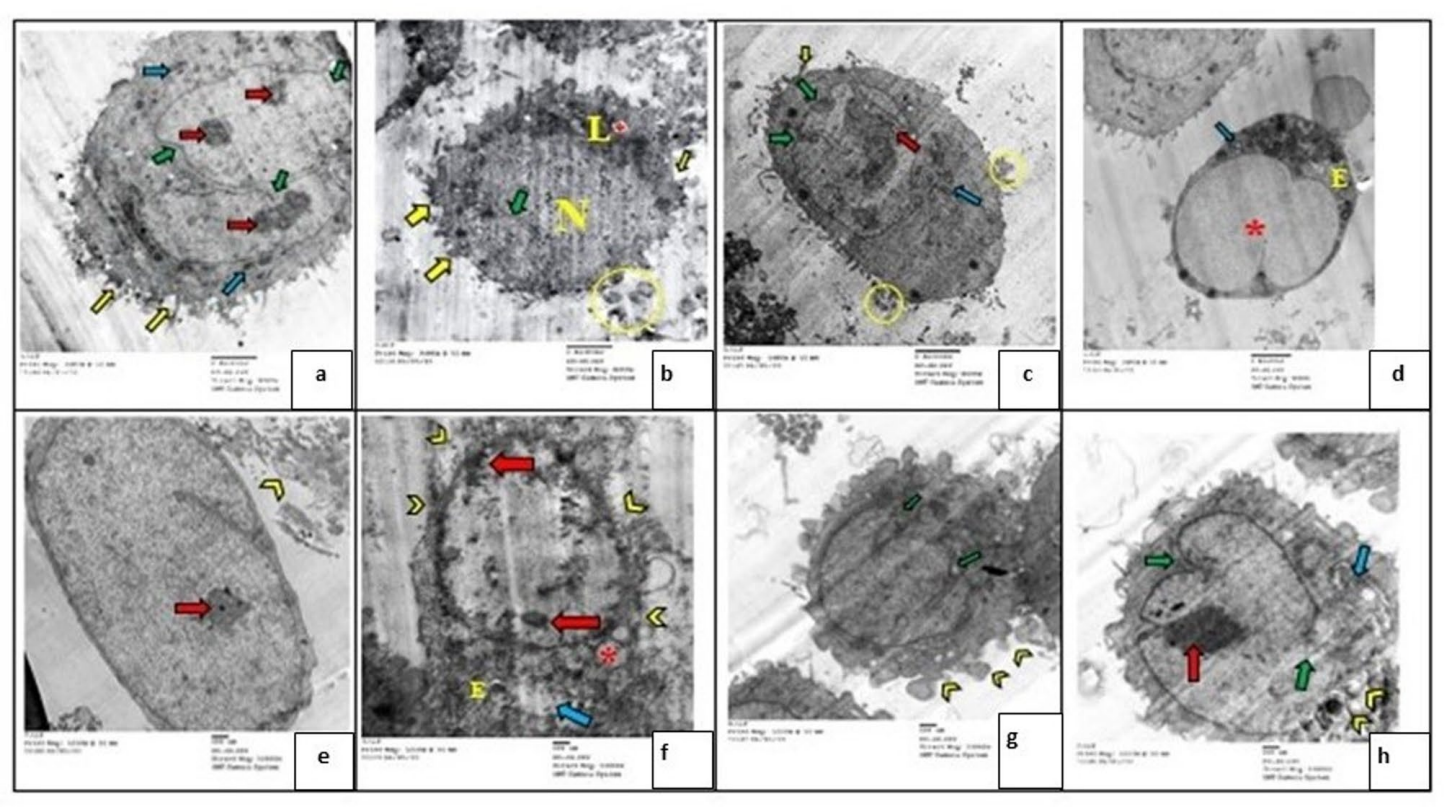
A photomicrograph of WI-38 cells showing: heterochromatic islands, marginalization and condensation of chromatin [red arrows], irregular nuclear membrane with focal fragmentation [green arrows], nuclear dissolution [N], membrane blebbing [yellow arrows], apoptotic bodies [yellow O], normal and swollen mitochondria [blue arrows], lysosomes [L], swollen RER [E], rupture of the plasma membrane [yellow arrow heads] and cytoplasmic vacuoles [*]. [**(a**,** b**,** c**,** d)** origin. mag X8000, **(e**,** f**,** g**,** h)** origin. mag X12000].

Evaluation of WI-38 fibroblast cells in different experimental groups by TEM showed different characterization figures of apoptosis and necrosis. Examination of TEM photomicrographs revealed cytoplasmic apoptotic features such as apparent cell shrinkage **(**Fig. 6b**)**, cell membrane blebbing **(**Fig. 6a, b, c**)**, apoptotic bodies **(**Fig. 6b, c**)**, cytoplasmic rarefaction **(**Fig. 6b**)**, some electron dense organelles (lysosomes) **(**Fig. 6b**)**, and few apparent normal mitochondria **(**Fig. 6a, c**).** While the nucleus showed apoptotic features such as heterochromatic islands **(**Fig. 6a, c**)**, marginal condensation of chromatin on the nuclear membrane **(**Fig. 6c**)**, an irregular nuclear membrane with focal fragmentations **(**Fig. 6a, b, c**)** and nuclear dissolution **(**Fig. 6b**).** On the other hand, some cells showed necrotic features such as swelling of RER **(**Fig. 6d), loss of mitochondrial structure **(**Fig. 6d, h**)**, cytoplasmic vacuoles **(**Fig. 6b, d, f**)**, perforation in cytoplasmic membrane and release of cellular content **(**Fig. 6f, g**)**, rarefaction and increase of nuclear cytoplasmic ratio **(**Fig. 6e, f, h**)**, few heterochromatic islands **(**Fig. 6e, f, h**)**, and folded nuclear membrane with nuclear margination of chromatin **(**Fig. 6e, f, g, h**).**

## Discussion

The leaves of MO are being extensively investigated for their potential therapeutic effects against various clinical conditions^28^. Notably, they are easily harvested and rich in resources, and contain active components such as flavonoids (myrecytin, quercetin and kaempferol), polyphenols^29^fatty acids, sterols, alkanes, phenylpropanoids, terpenoids, as well as vitamins and minerals^30^.

In the past decade, several researchers reported that MOLE demonstrated proliferative effect on normal fibroblast cell lines. Bioactive compounds isolated from the methanolic fraction of leaf extract have been correlated with enhanced proliferation and wound healing^31,32^. Additionally, the phenolic fraction exhibited no significant inhibitory effects on normal human dermal fibroblasts even with different concentrations^33^. Furthermore, it was stated that WI-38 cell lines showed a relatively high resistance to cytotoxicity against all tested nanoparticles of MOLEs^34^. Thus, this study was performed to assess the potential effect of MOLE on WI-38 cell line treated with EC e-liquid.

EC e-liquid has been shown to exhibit cytotoxic effects on normal human oral fibroblasts^23,25^as well as human oral keratinocytes, while stimulating the growth of cancerous keratinocyte cells. In addition, prolonged exposure to EC e-liquid reduced cell viability, consequently delaying the wound healing process^25^. This came in accordance with our findings, which revealed that EC e-liquid demonstrated a cytotoxic effect on WI-38 cells when compared to the untreated control cells. In addition, MOLE showed a decrease in cell viability in EC e-liquid treated cells.

It has been proposed that ECs are less harmful than conventional tobacco products due to their lower concentration of carcinogens and lower total number of chemicals^26^. However, the manufacturing process for e-liquids is not yet standardized, raising concern about the potential presence of carcinogenic substances in the diverse variety of e-liquids available on the market^35^. So far, several harmful chemical compositions have been identified in liquids and aerosols of ECs including glycol, nitrosamines, formaldehyde, acetaldehyde, chromium, nickel, aluminum, lead, silicon, and cadmium^36–39^.

To examine the effect of MOLE and to determine whether changes in cell cycle distribution contributed to the decrease in cell viability, analysis of apoptosis and cell cycle was performed in the present study using flow cytometric assay. Our findings revealed that MOLE induced apoptotic and necrotic cell death in the EC e-liquid treated WI-38 cells, along with cell cycle arrest at the S phase, interestingly exerting an anti-proliferative effect on EC e-liquid treated WI-38 cells.

It was previously stated that MOLE inhibited the proliferation of malignant and metastatic melanoma cell lines in a dose-dependent manner^33^Additionally, these extracts exhibited noticeable cytotoxicity in breast cancer cell line, particularly at higher concentrations^28^. These anti-proliferative effects of MOLE are likely due to its bioactive compounds, such as flavonoids that exhibit anti-proliferative properties and can promote cell cycle arrest at various phases^40–42^. Quercetin, a flavonoid found in MOLE, has been shown to induce apoptosis by activating the p53 tumor suppressor protein^43^. Additionally, MO extracts produce reactive oxygen species (ROS) that are target-specific, making them potent inhibitory agents as well^44^.

The cell cycle is a meticulously orchestrated sequence of events that take place in a cell leading to its division, and its regulation involves processes that are crucial to cell survival^45^. It is a four-stage process: the G1, S, G2, and M (mitosis) phases. Each phase is governed by the cyclins and cyclin-dependent kinases (CDKs)^46^. Cyclin-dependent kinase inhibitors (CDKIs) and tumor suppressor protein, p53, are known to regulate the activities of the cyclin/CDK complexes^47^. The S phase is the stage during which DNA synthesis and histone synthesis take place. Intra-S phase checkpoint turns off Cdk2 in response to DNA damage and blocks origin firing to avoid replication of damaged DNA^45^. In this connection, p53 is known to trigger apoptosis and arrest the cell cycle at the S and G2/M phases following DNA damage^48,49^. Meanwhile, p53 up-regulates multiple downstream-targeted genes, such as p21 and Bax^50^.

To further investigate the mechanism through which MOLE induced apoptotic cell death, we evaluated the expression of Bax and Bcl2, the main proteins involved in the regulation of apoptosis, using ELISA technique. Our findings showed that MOLE induced apoptosis through activating the apoptosis signaling intrinsic pathway by increasing the expression of the pro-apoptotic Bax while suppressing the expression of the anti-apoptotic Bcl2, altering the ratio of pro-apoptotic proteins to the anti-apoptotic proteins in favor of apoptosis, suggesting the mechanism through which it exerts its anti-proliferative activity in EC e-liquid treated WI-38 cell line.

The statistical analysis of Bax, Bcl2 and Bax/Bcl2ratio expression in the present study showed that the differences between groups were statistically significant. In accordance with our results, the anti-proliferative activity of MOLE through induction of apoptosis via up-regulation of Bax and down-regulation of Bcl2 was reported in various cell lines^51,52^.

The lack of cell death, which is characteristic of cancer, results from over-expression of anti-apoptotic genes and under-expression of pro-apoptotic genes. An unbalanced Bcl2/Bax ratio (Bcl2/Bax > 1) has been recognized in several studies as a signature of the acquisition of apoptosis resistance in cancer cells. Therefore, induction of apoptosis is considered an important objective of several anti-tumor and radio-sensitization drugs^53,54^.

## Materials and methods

### Material

- **Mammalian cell lines: WI-38** (normal human lung fibroblast cell line) was obtained from the American Type Culture Collection (ATCC, Rockville, MD).
- ***Moringa oleifera*** **leaves extract (MOLE)**: lyophilized MOLE was prepared in the National Research Center, Cairo, Egypt. Voucher specimens were deposited in the herbarium of the National Research Center; voucher no. MO-522 (*Moringa oleifera* L.) Giza, Egypt^16^.
- **EC e-liquid: A** high-grade formula of e-liquid with a strong grape flavor (VG/PG-50/50, Nicotine 12 mg) was purchased from Mazaj online store.
- **Chemicals and reagents**: Dulbecco’s modified Eagle’s medium (DMEM), fetal bovine serum, gentamycin, 0.25% trypsin-EDTA, HEPES buffer solution and L-glutamine were purchased from Lonza (Belgium). MTT dyes and dimethyl sulfoxide (DMSO) were purchased from Sigma (St. Louis, Mo., USA).

### Methods

#### Grouping

**Fig. 7:**
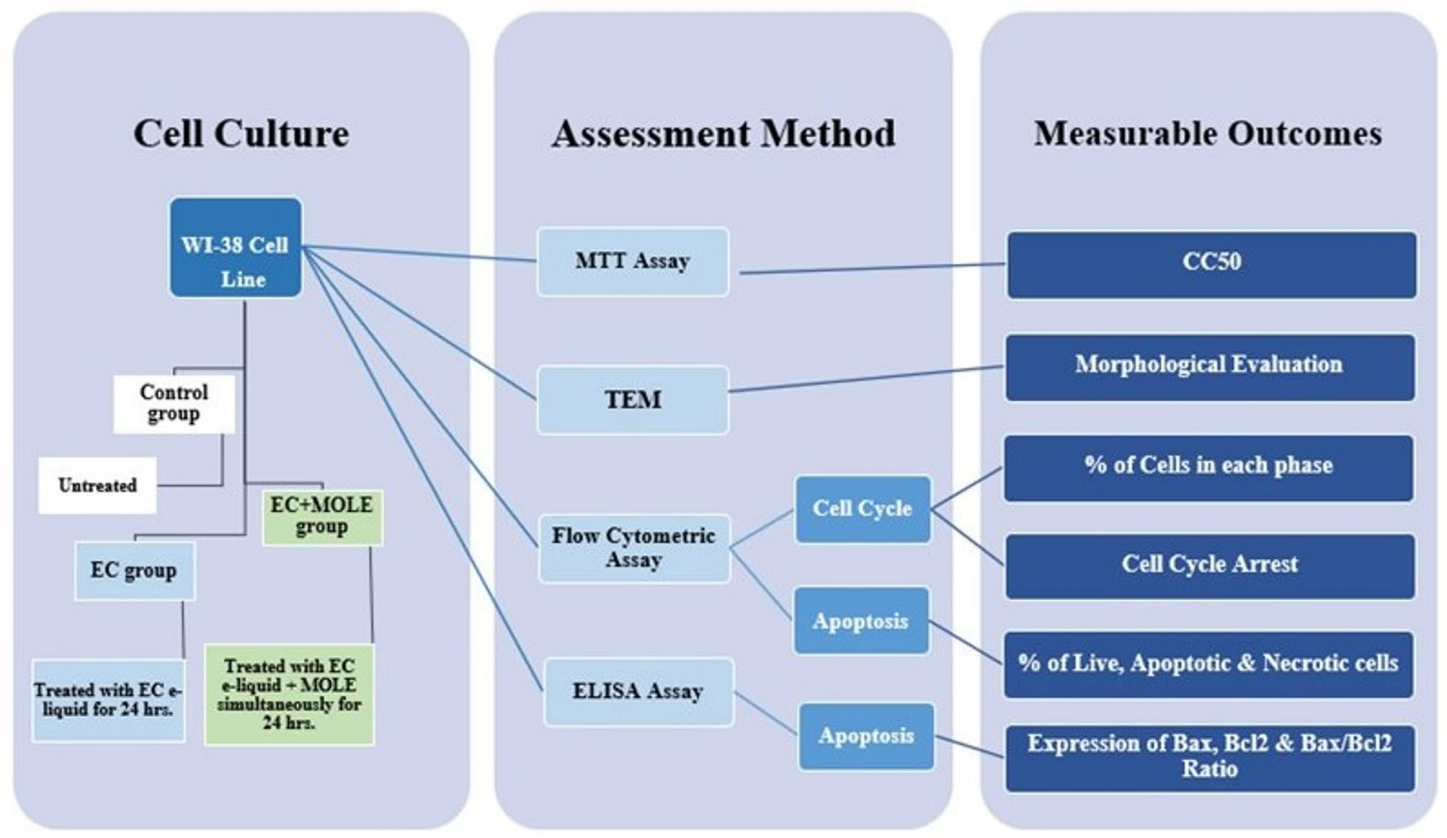
Schematic diagram illustrating the study design.

The research protocol was approved by the Faculty of Oral and Dental Medicine’s ethics committee at Future University in Egypt **(FUE.REC (33)/10-2023)**. WI-38 cell lines were divided into three groups. Control group received no treatment, EC group was treated with EC e-liquid only and EC + MOLE group received EC e-liquid and MOLE simultaneously for 24 h (Fig. 7).

#### Extract preparation

After obtaining the required permission for collecting MO leaves from National Research Center gardens and using them in various medicinal preparations under the supervision of the National Research Center, Giza, Egypt, voucher specimens, voucher no. MO-522 (*Moringa oleifera* L.), were deposited in the herbarium of the National Research Center, Giza, Egypt. Plants were authenticated by Prof. Mostafa El-Missiry, professor of Phytochemistry and Plant Systematics, National Research Center, Giza, Egypt. Field studies and experimental research on plants (either cultivated or wild), including the collection of plant material, complied with relevant international, national and institutional guidelines and legislation. MO leaves were collected, dried and ground after being carefully washed for 15 min under running water. Thereafter, for extract preparation, the ground leaves were immersed in 80% ethyl alcohol. The alcohol was evaporated using a rotary evaporator at 45 °C under reduced pressure^14,16^.

#### Cell line propagation

The cells were cultured in DMEM containing 10% heat-inactivated fetal bovine serum, HEPES buffer, 1% L-glutamine, and 50 µg/mL gentamycin. They were kept at 37 °C in a humidified environment with 5% CO2 and were sub-cultured twice a week^55^.

##### Assessment of the effect of EC e-liquid and MOLE on WI-38 cell line

###### 3-(4,5-Dimethylthiazol-2-yl)-2,5-diphenyltetrazolium bromide (MTT)assay

WI-38 cells were seeded in a 96-well plate at a density of 1 × 10⁴ cells per well in 100 µL of growth medium. After 24 h, fresh medium containing various concentrations (500 µg/mL, 250 µg/mL, 125 µg/mL, 62.5 µg/mL, 31.25 µg/mL, 15.6 µg/mL, and 7.8 µg/mL) of EC e-liquid and EC e-liquid combined with MOLE was added. Serial two-fold dilutions of the test compound were added to confluent cell monolayers in 96-well, flat-bottomed microtiter plates (Falcon, NJ, USA) using a multichannel pipette. Each concentration was tested in triplicate wells. After 24 h of incubation, the number of viable cells was determined by the MTT assay. Control cells were incubated without the test sample, with or without DMSO. An 85 µL aliquot of the medium was removed from each well, and 50 µL of DMSO was added and mixed thoroughly. Optical density was measured at 590 nm using a microplate reader (SunRise, TECAN, Inc., USA) to assess cell viability. The percentage of viability was calculated as [ODt/ODc) × 100%], where ODt is the mean optical density of treated wells and ODc is the mean optical density of untreated wells. Finally, the plates were inverted to remove the medium, the wells were washed three times with 300 µL of phosphate-buffered saline (pH 7.2), and the cells were fixed with 10% formalin for 15 min at room temperature^56–58^.

##### Morphological assessment of cytotoxicity against WI-38 cell line

For cellular morphology assessment, WI-38 cells were seeded in a 96-well plate at a density of 1 × 10^4^ cells per well and treated with different concentrations (500 µg/mL, 250 µg/mL, 125 µg/mL, 62.5 µg/mL, 31.25 µg/mL, 15.6 µg/mL and 7.8 µg/mL) of EC e-liquid and EC e-liquid and MOLE) for 24 h. For observing cellular morphology, an inverted microscope (CKX41; Olympus, Japan) equipped with a digital microscopy camera was used. Images representing the morphological changes were captured and compared to control cells at 100x.

##### Flow cytometric assays

###### Apoptosis analysis (Annexin V-FITC/PI assay) using flow cytometry

Apoptosis was analyzed using the Annexin V-FITC/PI double staining assay. Briefly, WI-38 cells were seeded in a 6-well plate at a density of 1 × 10⁶ cells per well, cultured to form a confluent monolayer, and treated with the test samples at their CC50 concentrations (190.2 µg/mL of EC e-liquid and 33.2 µg/mL of EC e-liquid with MOLE). After 24 h of treatment, the cells were harvested and rinsed twice in PBS for 20 min each, followed by binding buffer. Treated and non-treated WI-38 cells were then re-suspended in 100 µL of kit binding buffer, and 1 µL of FITC-Annexin V (Becton Dickinson BD PharmingenTM, Heidelberg, Germany) was added, followed by incubation at 4 °C for 40 min. The cells were then washed, re-suspended in 150 µL of binding buffer, and 1 µL of PI (1 µg/mL in PBS) (Invitrogen, Life Technologies, Darmstadt, Germany) was added. Finally, the cells were analyzed using a BD FACS Calibur flow cytometer (BD Biosciences, San Jose, CA)^59^.

###### Cell cycle analysis using flow cytometry

The CycleTEST^™^ PLUS DNA Reagent Kit (Becton Dickinson Immunocytometry Systems, San Jose, CA) was used for cell cycle analysis. WI-38 cells were seeded in a 6-well plate with a concentration of 1 × 10^6^ cells per well. Cells treated with the tested samples (190.2 µg/mL of EC e-liquid and 33.2 µg/mL of EC e-liquid and MOLE) or non-treated were stained with propodium iodide stain following the procedure provided by the kit and then run on the cytometer. Cell Quest software (Becton Dickinson Immunocytometry Systems, San Jose, CA) was used to evaluate the cell cycle distribution^59^.

##### Enzyme-linked immunosorbent assay (ELISA)

WI-38 cells (treated or non-treated) were further examined for apoptotic and anti-apoptotic markers. ELISA colorimetric kits were used for the analysis of the apoptotic marker Bcl2-associated X protein gene (Bax) levels as well as the anti-apoptotic marker B cell lymphoma/leukemia-2 gene (Bcl2) levels according to the manufacturer’s instructions. After 24 h of treatment, the cells were harvested through trypsinization by being digested with trypsin (0.25%), and collected after centrifugation for 5 min, then rinsed twice in PBS (20 min each) followed by binding buffer^60^.

##### Transmission electron microscope (TEM) examination

At the Regional Center for Mycology and Biotechnology (RCMB), Cairo, Egypt, WI-38 cells were centrifuged at 2000×g for 10 min. The cell pellets were fixed in 3% glutaraldehyde in 0.1 M sodium cacodylate buffer (pH 7.0) for 2 h, rinsed, and fixed in 1% osmium tetroxide at room temperature for 2 h. Samples were dehydrated in ethanol series ranging from 10 to 100% for 15 min in each alcohol dilution and lastly with absolute ethanol for 30 min. Samples were eventually fully penetrated with pure resin through a graded sequence of epoxy resin and acetone infiltrations. Ultra-thin sections were collected on copper grids. The sections were then dyed twice with uranyl acetate and lead citrate and examined at 80 kV using a JEOL- JEM 1010 transmission electron microscope^61^.

##### Statistical analysis

Recorded data were analyzed by IBM SPSS version 27 for performing the statistical analysis. Mean ± standard deviation (SD) was employed to express continuous variables. In addition, for each selected variable, box plots were created for descriptive purposes to determine the presence of any significant difference in apoptosis among the treatment groups per time point. The Kruskal–Wallis test was performed as a non-parametric multiple comparison test. P-values were reported, and a p-value < 0.05 was considered statistically significant.

## Conclusion

In conclusion, the present study revealed that MOLE demonstrated an inefficient cytoprotective effect on WI-38 cells against EC e-liquid cytotoxicity; however, it demonstrated an anti-proliferative effect through induction of apoptosis via up-regulation of Bax and down-regulation of Bcl2. Moreover, the S phase cell cycle arrest contributed to the anti-proliferative effect of MOLE. Hence, MOLE could be highly beneficial as an anti-proliferative agent.

## Data Availability

The data that Support the findings of this study are available from the corresponding author upon request.
